# Supplementary material for: A mixed culture of bacterial cells enables an economic DNA storage on a large scale
Source: Commun Biol. 2020 Jul 31;3:416. doi: 10.1038/s42003-020-01141-7 (PMC7395121; doi:10.1038/s42003-020-01141-7)
Supplement: Supplementary file 1 — Supplementary Information [file 42003_2020_1141_MOESM1_ESM.pdf]

SUPPLEMENTARY INFORMATION

Supplementary Figures

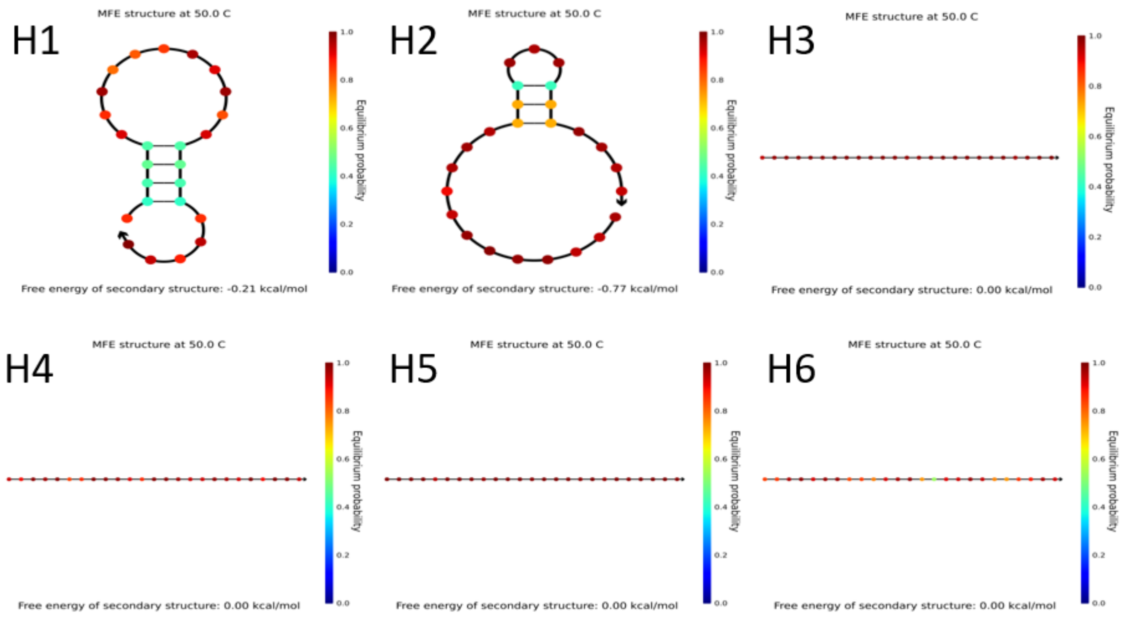

Supplementary Figure 1. Thermodynamic secondary structure in designed homologous arm sequence calculated by NUPACK (http://www.nupack.org). H1, H2 is the homologous arm with sequence from vector plasmid pUC19; H3, H4, H5 and H6, are the in silico designed sequence for homologous assembly.

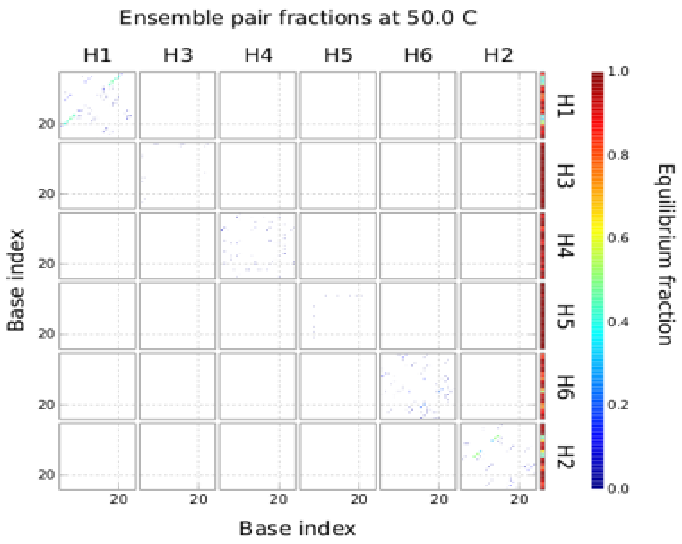

Supplementary Figure 2. Thermodynamic diagram for cross interaction between designed homologous arm sequence assessed by NUPACK. H1, the left homologous arm on the pUC19; H2, the right homologous arm on the pUC19; H3, H4, H5 and H6, the designed-homologous arms.

a

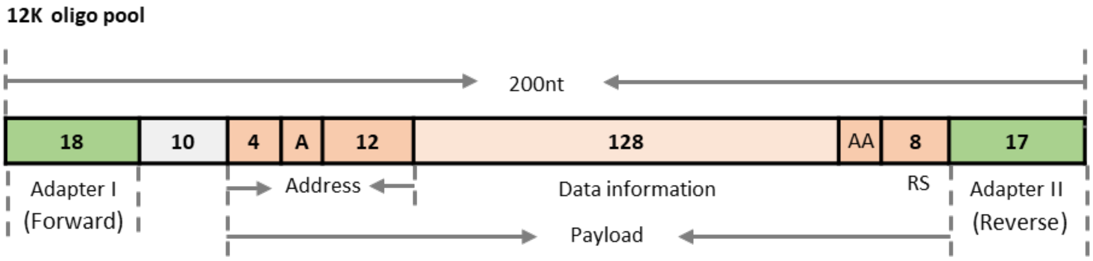

b

Serial number	Avoided Sequences	Type
1	GCGGCCGC	Not I
2	AGATAG	Primer
3	TGTTGG	Primer
4	GAGCTG	Primer
5	AGTCTG	Primer
6	AAAAA	Poly A
7	TTTT	Poly T
8	GGGG	Poly G
9	CCCC	Poly C

c

Data	File Size	Number of DNA strands
Central dogma (.jpg)	35 KB	990
DNA helix (.gif)	81 KB	2292
China Classical literature (.txt)	164 KB	4641
A Brief History of Element (.txt)	34 KB	962
Panda burn incense (.rar)	66 KB	1867
Human Mitochondrial	65 KB	768
Total	445KB	11520

Supplementary Figure 3. The information of 11520 oligos pool.

(a) Structure of the oligos unit designed under the same principle of our previous study, and synthesized from chip-based 12K oligo synthesis product of Twist Bio. (b) Sequences were avoided in the process of encoding. (c) The list of 445 KB digital files encoded in the 11520 oligos pool.

primer	Sequence		
	Homologous arm	Not I	Primer
TY-primer 1-F	TATCCCCTGATTCTGTGGATAACCG	GCGGCCGC	TGCATCACCTACCTCAGC
TY-primer 1-R	ACCTAACAAACCCAACAAACCCAAG	GCGGCCGC	TCCACGACGATCAGACT
TY-primer 2-F	CTTGGGTTTGTTGGGTTTGTTAGGT	GCGGCCGC	TGCATCACCTACCTCAGC
TY-primer 2-R	GTTATCCGGTCTTGCTTTACTCTGT	GCGGCCGC	TCCACGACGATCAGACT
TY-primer 3-F	ACAGAGTAAAGCAAGACCGGATAAC	GCGGCCGC	TGCATCACCTACCTCAGC
TY-primer 3-R	TATAAAAATAGGCGTATCACGAGGC	GCGGCCGC	TCCACGACGATCAGACT

Supplementary Figure 4. The primer sequence for 11520 oligos pool insert fragment construction. Homologous arm sequence was indicted in black, *Not* I cleavage site in blue and primer sequence in purple (forward) and orange (reverse).

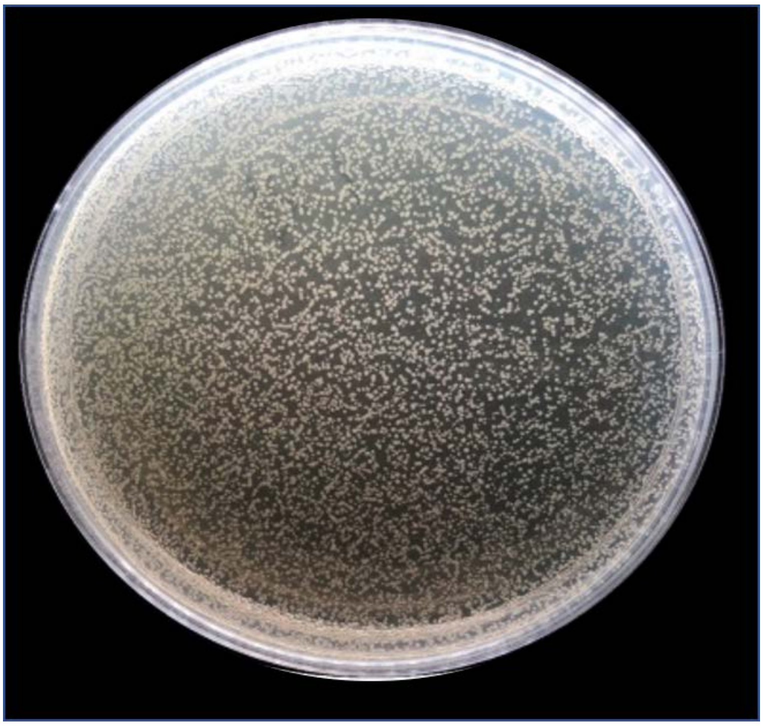

Supplementary Figure 5. One petri-dish of solid medium for one insert fragment (1F) assembly of 509 oligos pool, the colony number was counted from all the solid medium plates.

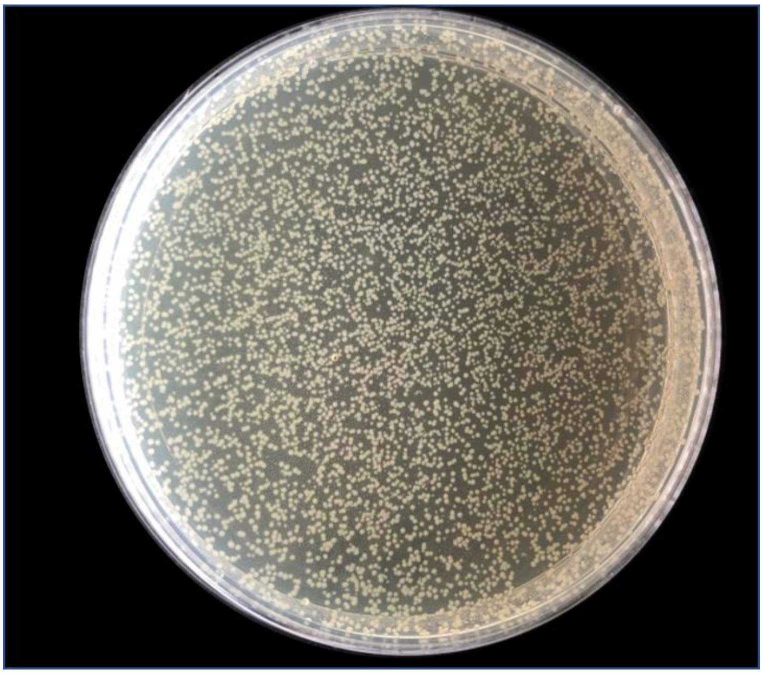

Supplementary Figure 6. One petri-dish of solid medium for one insert fragment (3F) assembly of 509 oligos pool, the colony number was counted from all the solid medium plates.

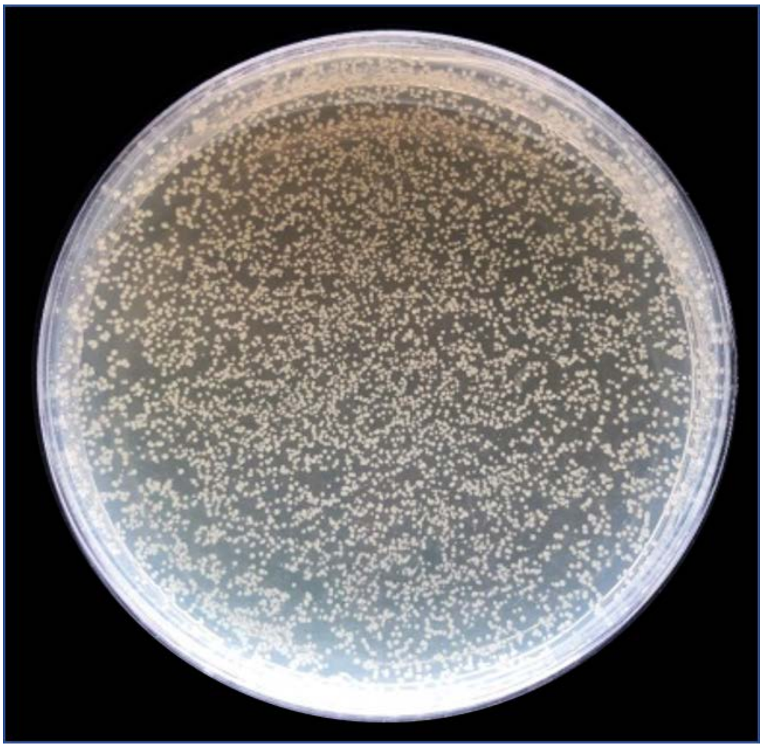

Supplementary Figure 7. One petri-dish of solid medium for one insert fragment (5F) assembly of 509 oligos pool, the colony number was counted from all the solid medium plates.

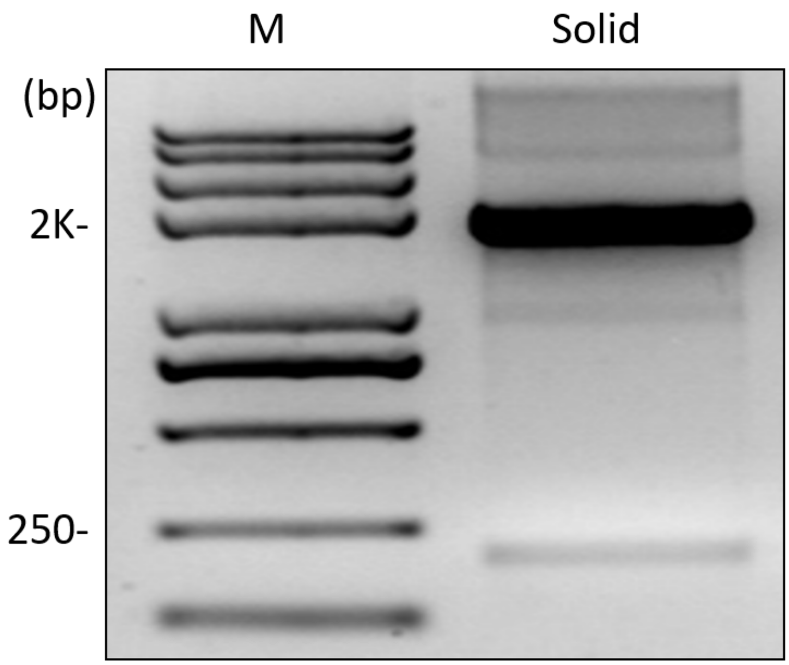

Supplementary Figure 8. Digestion of 1F assembly plasmids by *Not* I (509 oligos pool). M, 2K plus II DNA marker; Solid, cultured on LB plate medium.

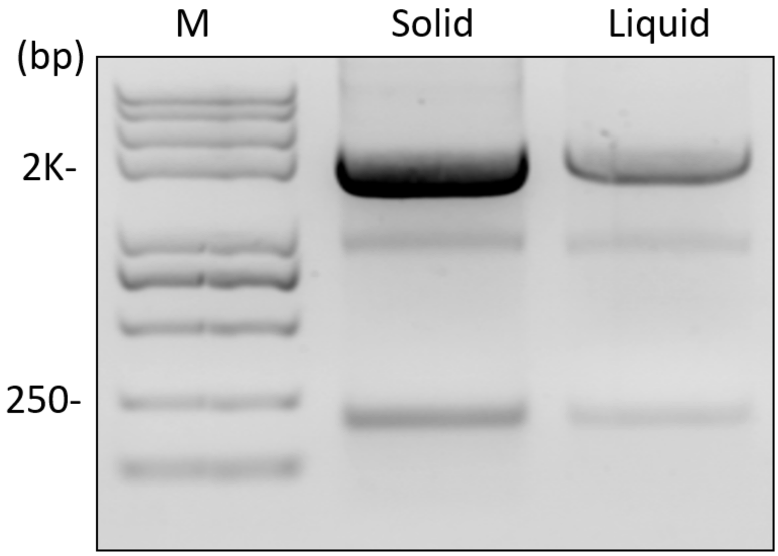

Supplementary Figure 9. Digestion of 3F assembly by *Not* I (509 oligos pool). M, 2K plus II DNA marker; Solid, cultured on LB plate medium; Liquid, cultured In LB liquid medium.

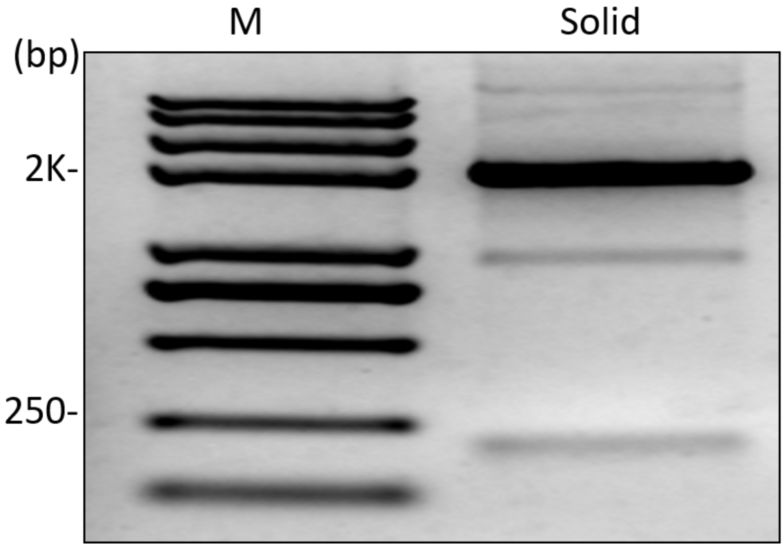

Supplementary Figure 10. Digestion of 5F self-assembly by *Not* I (509 oligos pool). M, 2K plus II DNA marker; Solid, cultured on LB plate medium.

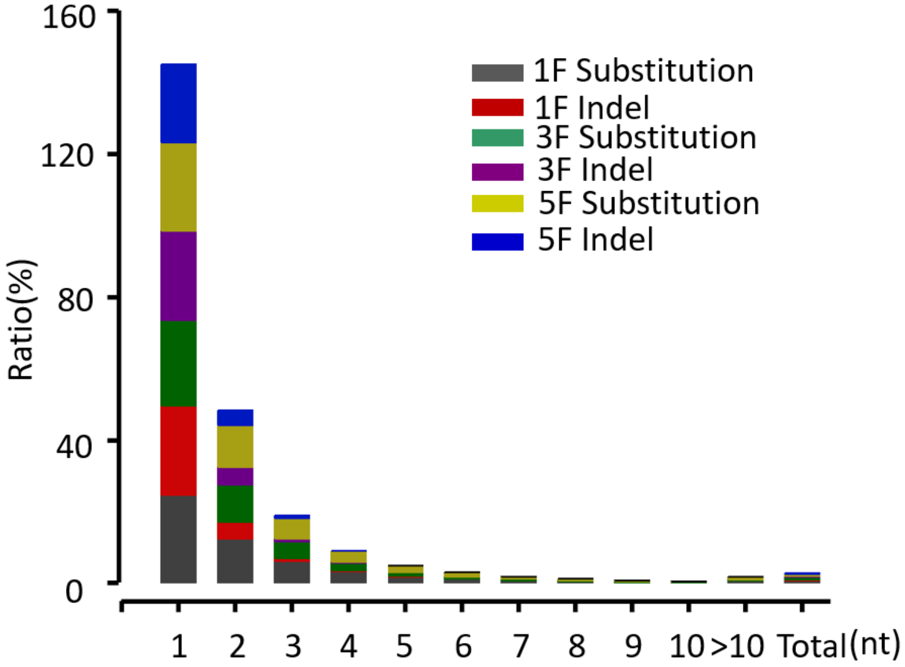

Supplementary Figure 11. Sequenced reads with base error (substitution or indel) were sorted out and the ration of reads with various number of base errors was calculated for assembly of 509 oligos pool.

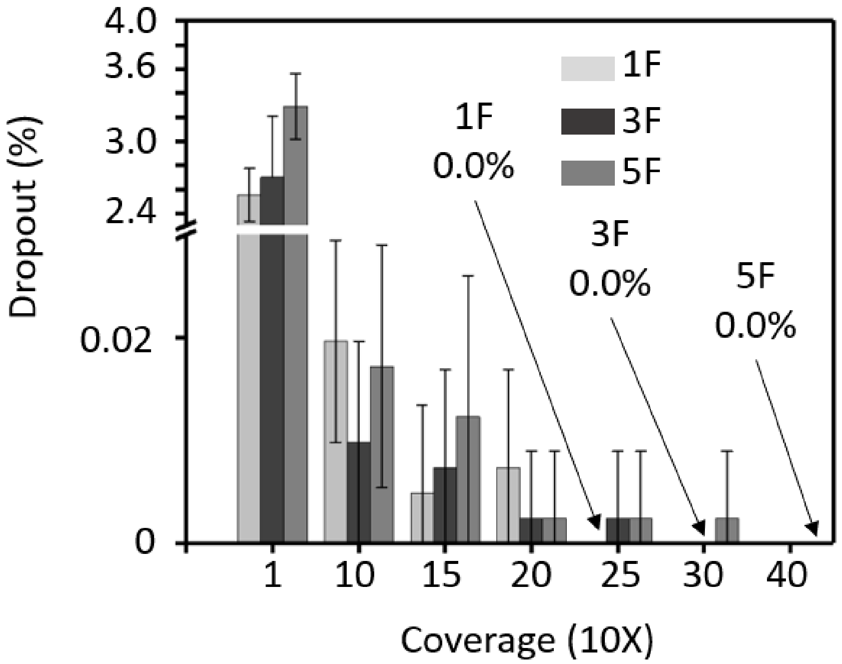

Supplementary Figure 12. The dropout of 1F, 3F and 5F assembly of 509 oligos pool in solid medium culture. When down-sampling the sequencing reads number to 250x of original oligo pool, the dropout of 1F was 0%; When down-sampling the sequencing reads to 300x, the dropout of 3F was 0%; When down-sampling the sequencing reads to 400x, the dropout of 5F was 0%. The position of 0% was indicated by arrow. Error bars represent the SD, where n =10.

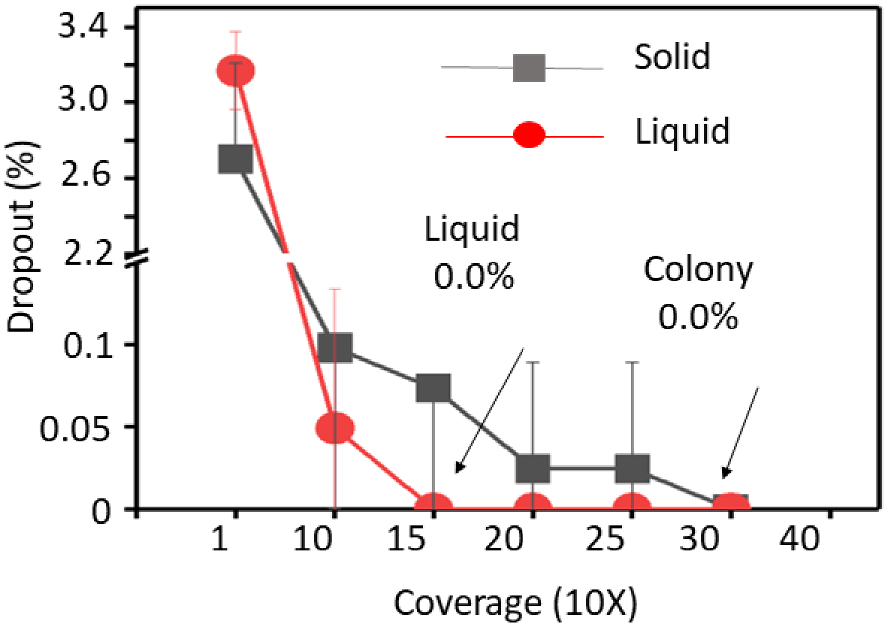

Supplementary Figure 13. The dropout of 3F assembly of 509 oligos pool in solid or liquid culture. When down-sampling the sequencing reads to 150x, the dropout of liquid culture was 0% and down-sampling the sequencing reads to 300x, the dropout of solid culture was 0%. The arrow indicates the position where dropout of each sample is 0 %. Error bars represent the SD, where n =10.

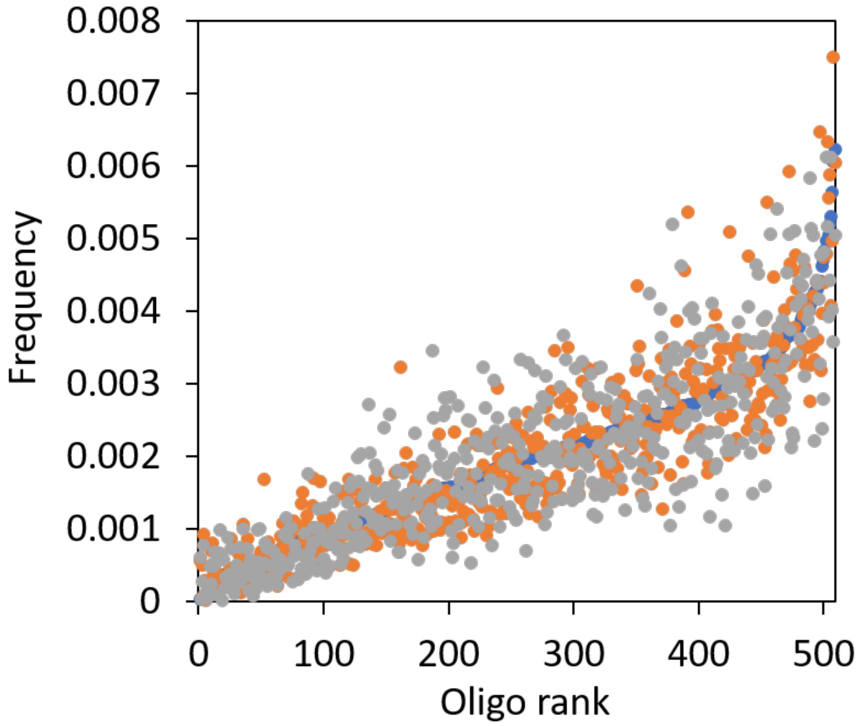

Supplementary Figure 14. Sequenced reads frequency of each 509 oligos reference, for one insert fragment (1F, Blue) assembly, three fragments (3F, orange) assembly and five fragments (5F, gray) assembly.

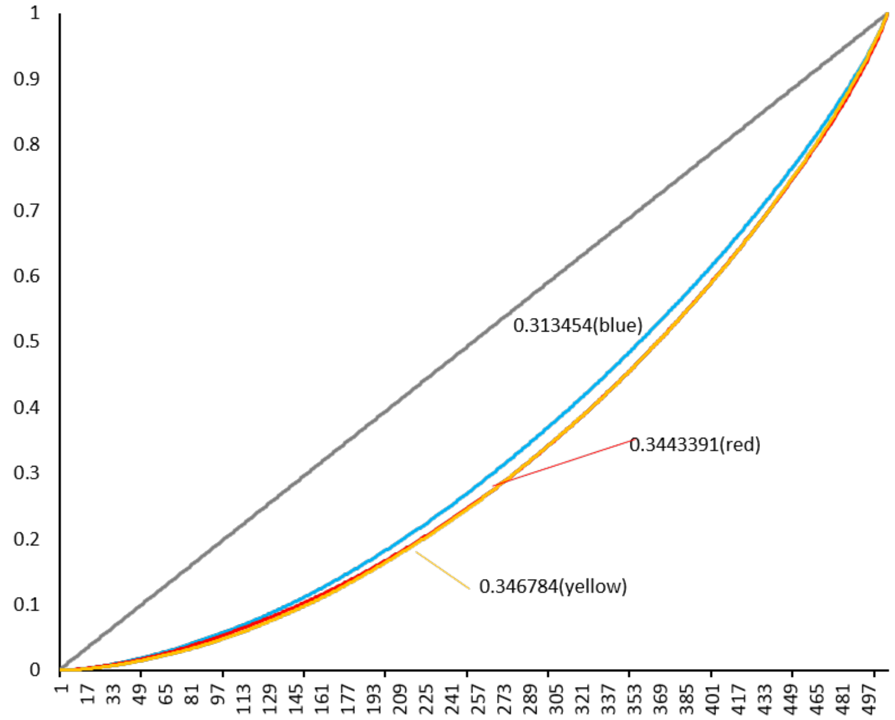

Supplementary Figure 15. Lorenz curve of Gini coefficient for recovered oligos of 1F (blue, with a Gini of 0.313454), 3F (Red with a Gini of 0.3443391) and 5F (Orange with a Gini of 0.346784) assembly of 509 oligos pool.

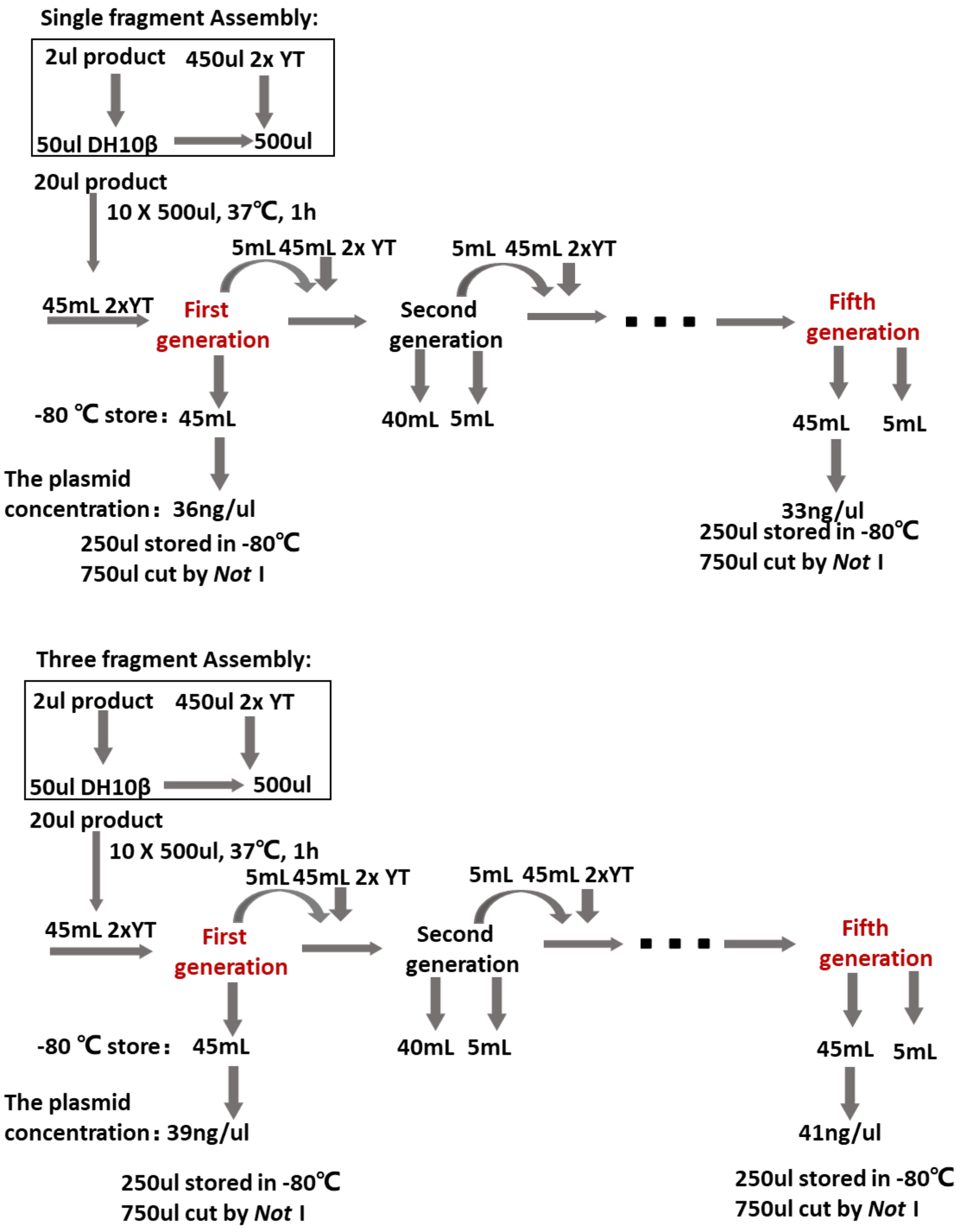

Supplementary Figure 16. The workflow for 11520 oligos pool assembly for mixed culture in liquid medium. The plasmid concentration indicates the extracted plasmid after culture.

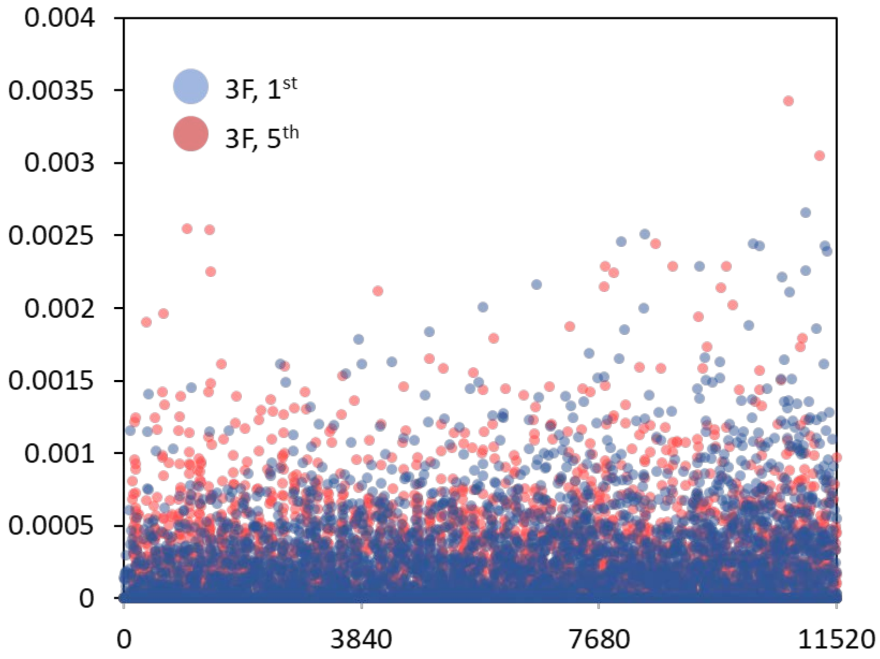

Supplementary Figure 17. The sequenced reads frequency of each 11520 oligos reference of the 1st passaging of three insert fragment (3F 1st, red dot) and the 5th passaging (3F 5th, blue dot).

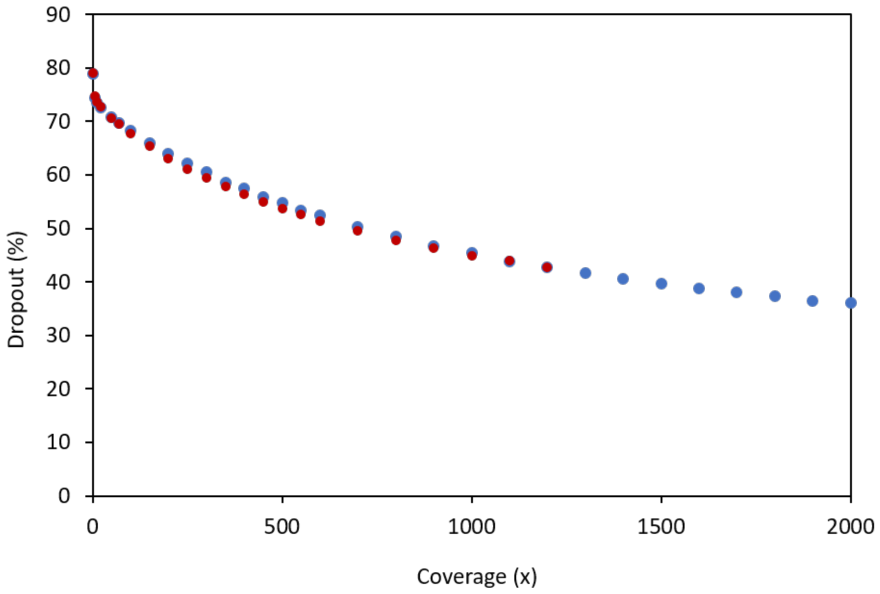

Supplementary Figure 18. Oligo dropout rate was plotted to the corresponding sequencing reads depth. The 1st passaging of three fragment assembly (3F 1st, blue) and the 5th passaging (3F 5th, red).

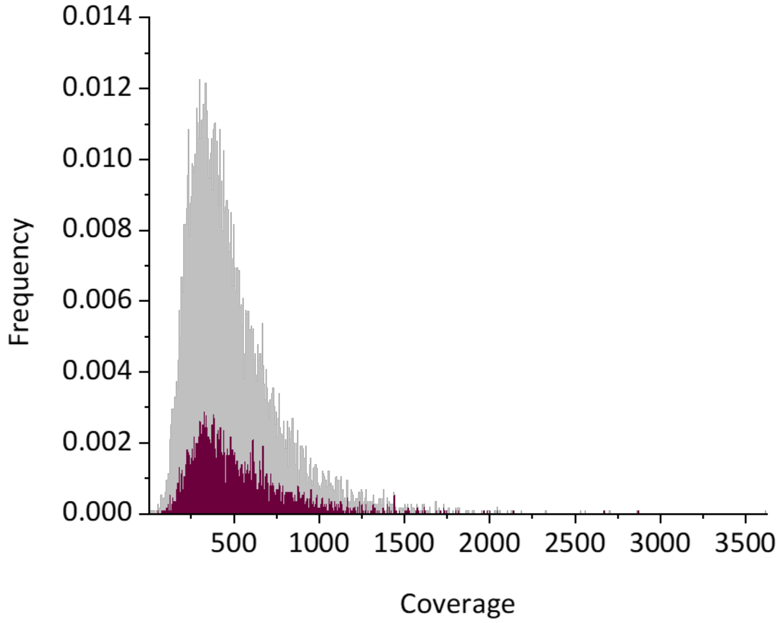

Supplementary Figure 19. The oligo group (red line) which dropout from the 1st passaging of three fragment assembly sample was mapped to the oligos frequency distribution of original master oligo pool (gray line).

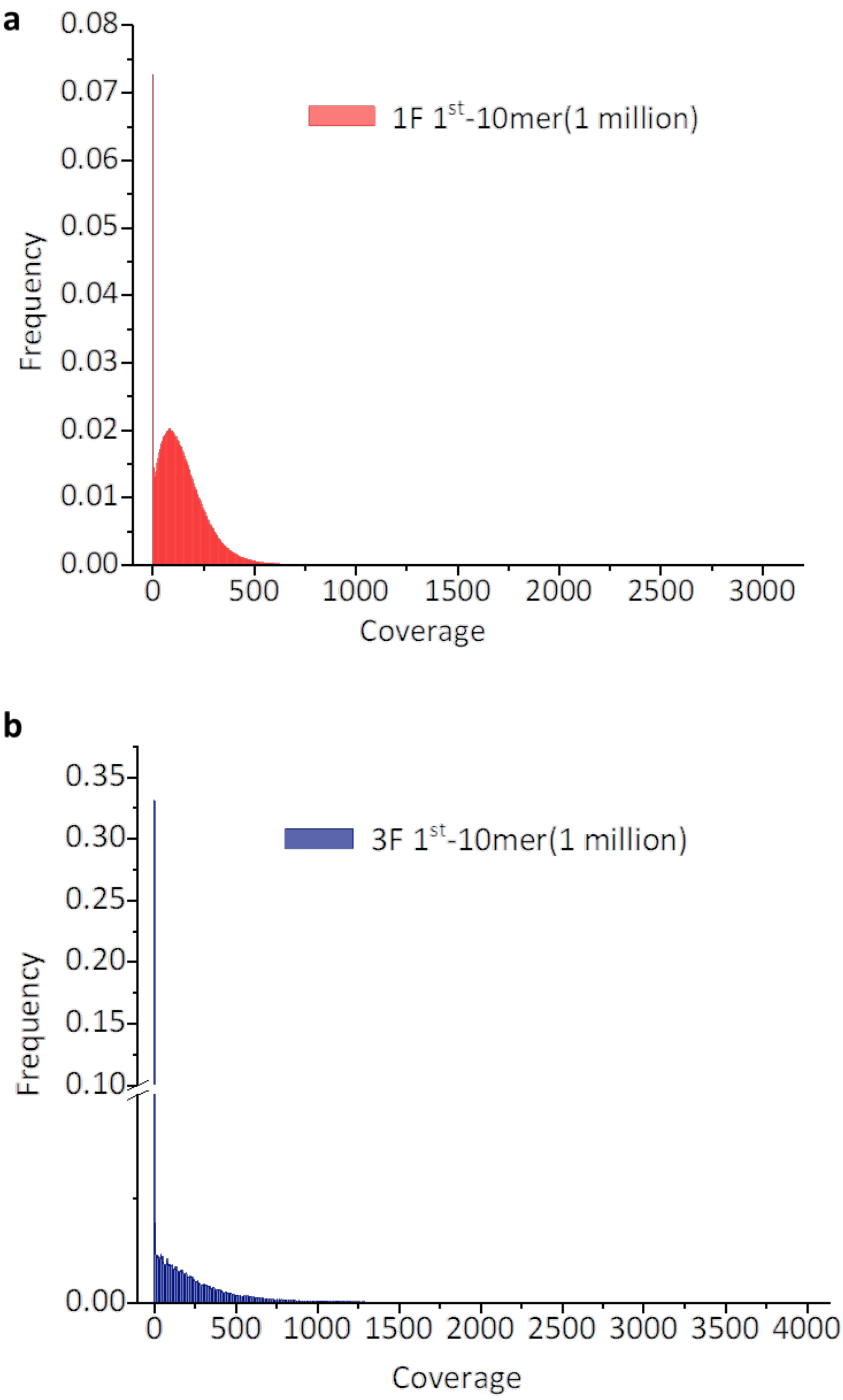

Supplementary Figure 20. 10-mers nucleotide pattern frequency distribution of 1 million valid sequencing reads from sample of 1F 1st (a) and 3F 1st (b).

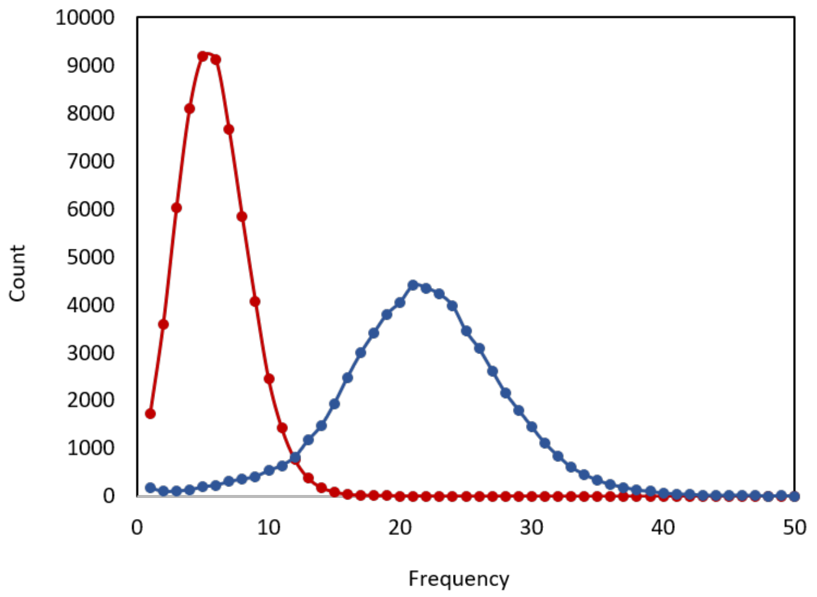

Supplementary Figure 21. 10-mers frequency of three fragment assembly of 11520 oligos pool. Red: enriched oligos, Blue: deprived oligos in comparing with 1F 1st.

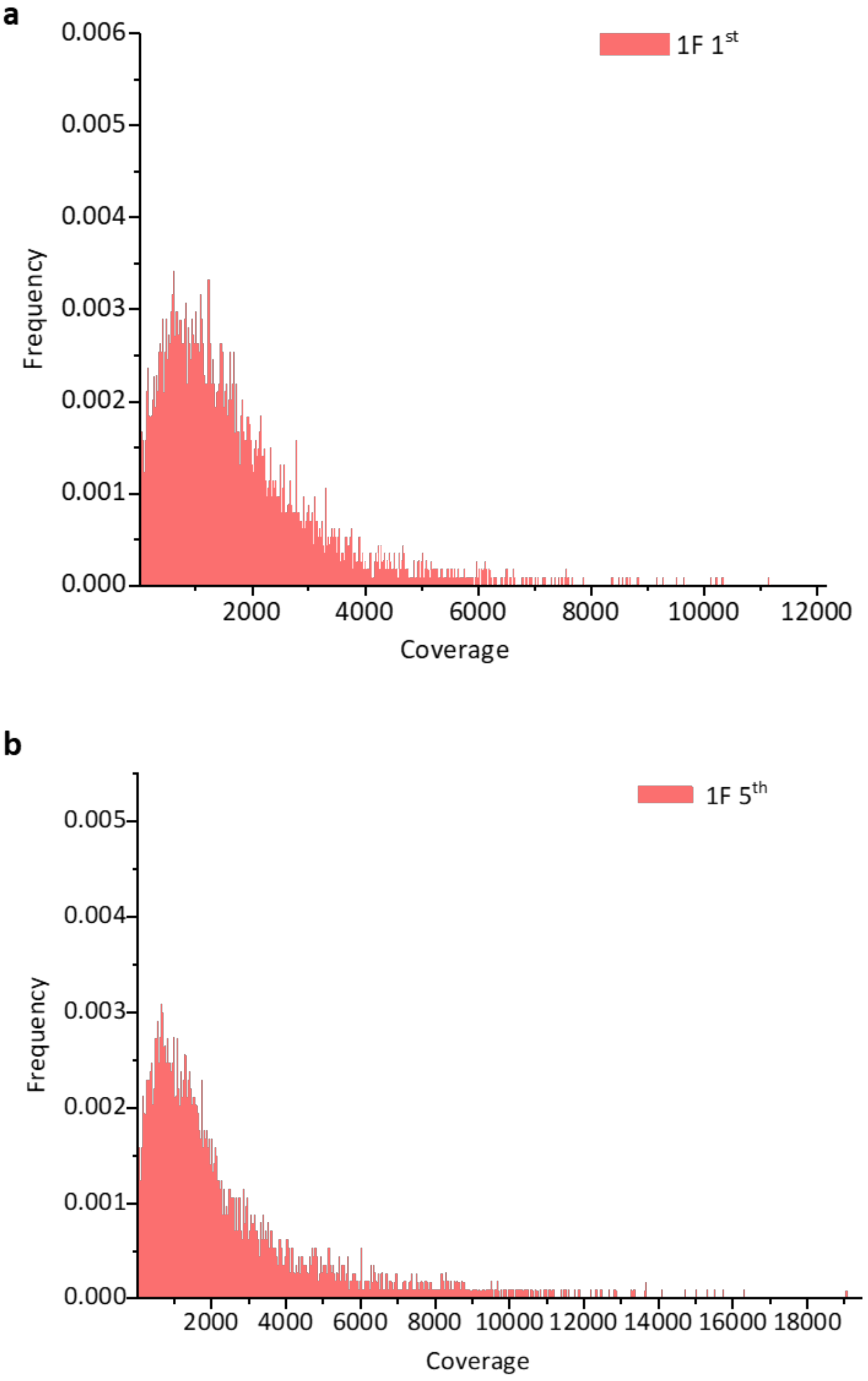

Supplementary Figure 22. Oligo frequency distribution of retrieved oligos from the 1st passaging of one insert fragment assembly (1F 1st, a) and the 5th passaging (1F 5th, b) of 11520 oligos pool.

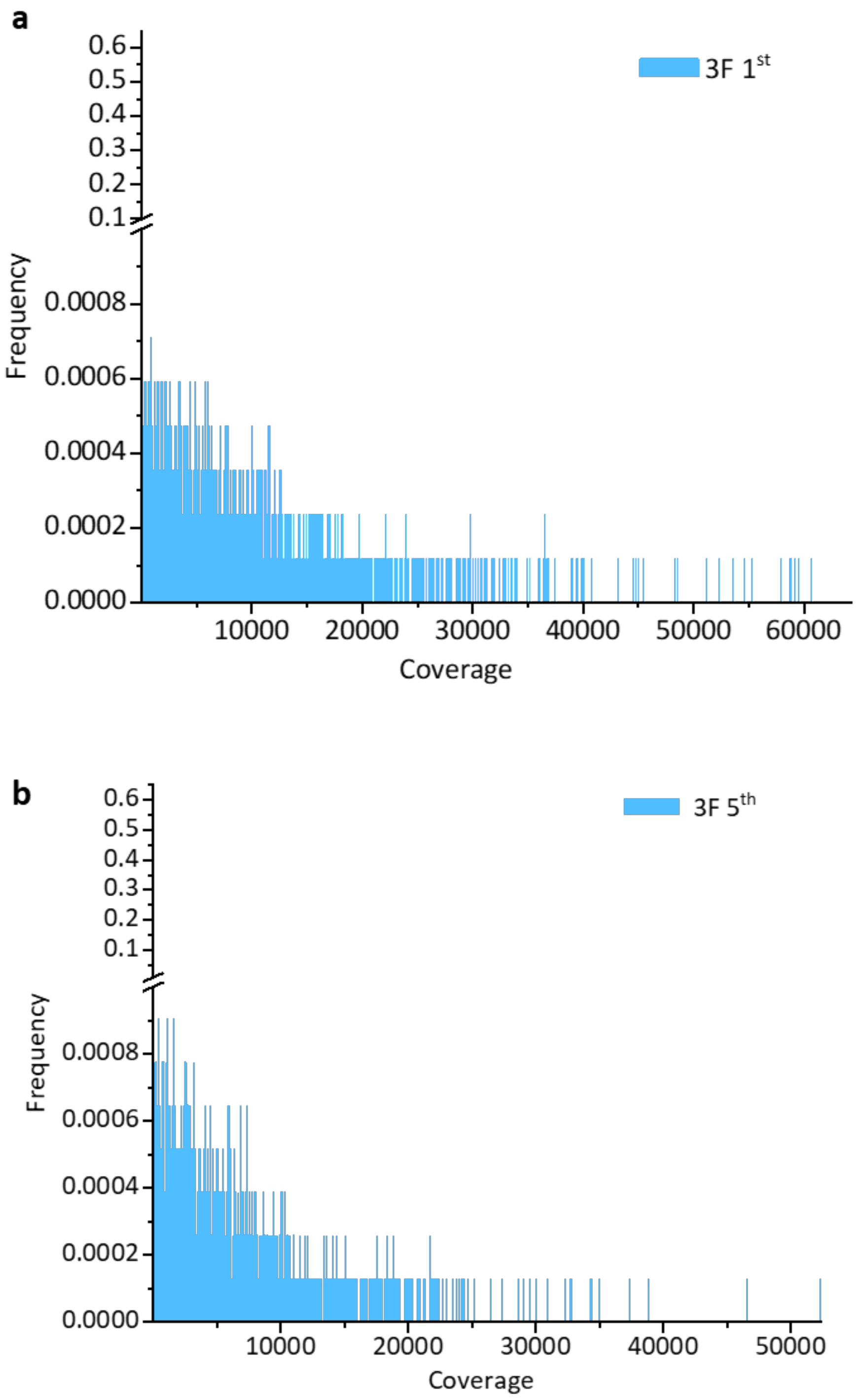

Supplementary Figure 23. Oligo frequency distribution of retrieved oligos from the 1st passaging of three insert fragment assembly (3F 1st, a) and the 5th passaging (3F 5th, b) of 11520 oligos pool.

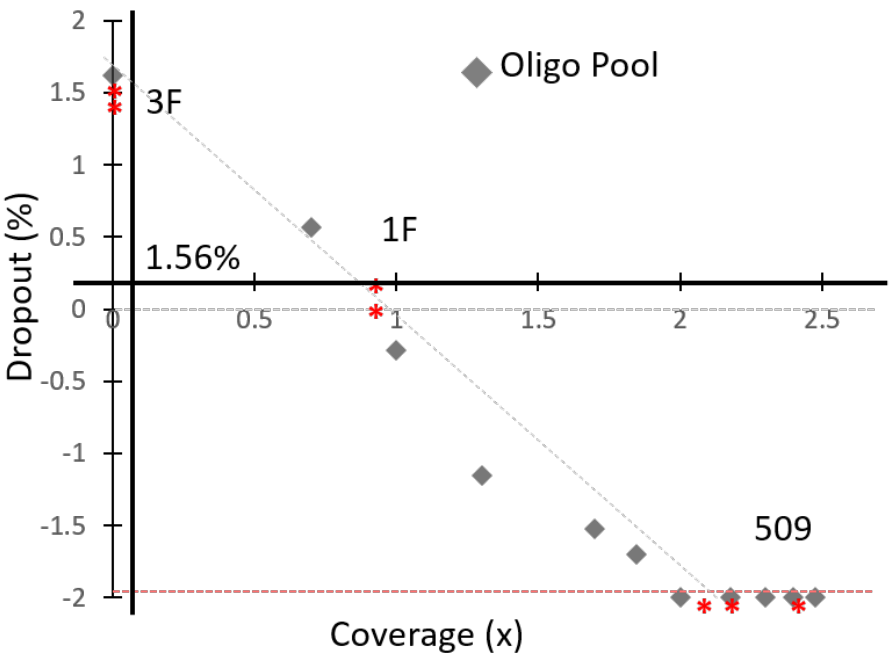

Supplementary Figure 24. Dropout was plotted to corresponding sequencing reads depth. The dropout rate of 11520 master pool was plotted to the corresponding sequencing reads depth (gray diamond) and the dropout rate (red star) of one fragment 11520 oligos assembly sample (1F), three fragments 11520 oligos assembly sample (3F) and all 509 oligos assembly sample respectively were mapped in the dropout rate curve.

Supplementary Tables

Supplementary Table 1. Sequence information of primers.

Primer name	Sequence (5'→3')	For oligo pool
F01	TCACCATCCACTCTAAACAC	509 oligos pool
R01	CACTTTACACCTCCACTCAT	
F02	ACCCTCACCTATCAACTCAA	
R02	CTTCCGACCACTATACCTCT	
F03	ACTCCCACTCACCTATATCC	
R03	ATAACCTCACTCACCTACCA	
F04	ACTCTCACCTTTACTCCCAC	
R04	CTACTCCCACTACTACCACA	
1-F	TATCCCCTGATTCTGTGGATAACCGGCGGCCGCACCCTCACCTATCAACTCAA	
1-R	ACCTAACAAACCCAACAAACCCAAGGCGGCCGCCTTCCGACCACTATACCTCT	
2-F	CTTGGGTTTGTTGGGTTTGTTAGGTGCGGCCGCACCCTCACCTATCAACTCAA	
2-R	GTTATCCGGTCTTGCTTTACTCTGTGCGGCCGCCTTCCGACCACTATACCTCT	
3-F	ACAGAGTAAAGCAAGACCGGATAACGCGGCCGCACCCTCACCTATCAACTCAA	
3-R	TCCCACACACCCACCCAACCTACAAGCGGCCGCCTTCCGACCACTATACCTCT	
4-F	TTGTAGGTTGGGTGGGTGTGTGGGAGCGGCCGCACCCTCACCTATCAACTCAA	
4-R	ATAGATTTCCATTACTCACCGCTTGGCGGCCGCCTTCCGACCACTATACCTCT	
5-F	CAAGCGGTGAGTAATGGAAATCTATGCGGCCGCACCCTCACCTATCAACTCAA	
5-R	TATAAAAATAGGCGTATCACGAGGCGCGGCCGCCTTCCGACCACTATACCTCT	
PCR-pUC19-F	GCCTCGTGATACGCCTATTT	
PCR-pUC19-R	CGGTTATCCACAGAATCAGG	
F1	TGCATCACCTACCTCAGC	11520 oligos pool
R1	TCCACGACGATCAGACT	
TY-primer 1-F	TATCCCCTGATTCTGTGGATAACCGGCGGCCGCTGCATCACCTACCTCAGC	

TY-primer 1-R	ACCTAACAAACCCAACAAACCCAAGGCGGCCGCTCCACGACGATCAGACT	
TY-primer 2-F	CTTGGGTTTGTTGGGTTTGTTAGGTGCGGCCGCTGCATCACCTACCTCAGC	
TY-primer 2-R	GTTATCCGGTCTTGCTTTACTCTGTGCGGCCGCTCCACGACGATCAGACT	
TY-primer 3-F	ACAGAGTAAAGCAAGACCGGATAACGCGGCCGCTGCATCACCTACCTCAGC	
TY-primer 3-R	TATAAAAATAGGCGTATCACGAGGCGCGGCCGCTCCACGACGATCAGACT	

Supplementary Table 2. The Gini Coefficient of each sample with 11520 oligos pool.

Sample	Gini Coefficient
Master Pool	0.29
1F 1st	0.41
1F 5th	0.48
3F 1st	0.87
3F 5th	0.87

Note: Gini coefficient of retrieved oligos for each assembled mixed culture of 11520 oligos pool, master pool is the original oligo pool from chip-based synthesis and amplified by PCR.

Supplementary Table 3. The data statistics for oligo retrieved from 1F or 3F with 11520 oligos pool by direct *Not* I digest or PCR amplification.

Sample	Dropout Rate	Coverage	Perfect Decoding
1F 1st *Not* I	0.90%	1472x	Yes
1F 5th *Not* I	1.40%	1900x	Yes
1F 1st PCR	0.40%	2928x	Yes
1F 5th PCR	1.40%	2399x	Yes
3F 1st *Not* I	26.50%	2101x	No
3F 5th *Not* I	32.80%	1325x	No
3F 1st PCR	25.00%	2797x	No
3F 5th PCR	71.70%	2720x	No

Note: The values of dropout rate in the gold box are less than 1.56%, corresponding samples can be decoded perfectly. The values in the light blue box are over 1.56% and less than 50%, they cannot be decoded. The value in the blue box is over 50%, the sample cannot be decoded.

Supplementary Notes

Supplementary Note 1. BASIC Code

Gene coding is a new type of distributed storage system. In this work, we use the BASIC Code, it is a kind of distributed erasure code designed for gene coding, aiming at maximizing storage utilization, effectively guaranteeing the reliability of the storage system. Due to the adjustable system parameters K and L, we take the standard system parameters (K =252, L =256) as an example. Here, we use 11,520 DNA sequences (12K oligo pool) of length 200 nt with payload of length 155 nt to store 445 KB data (Supplementary Fig. 3).

Supplementary Note 2. Encoding and Decoding Process

Encoding process. The goal of this work is to transform the input file to DNA sequence reads (within biochemical constraints). DNA BASIC Code should enable error-detection, error-correction and full recovery. There are mainly steps: (a) erasure coding, (b) RS coding. (c) filtering. Since the sequence reads need to satisfy the biochemical constraints, both (a) and (b) include the step of filtering the sequences.

Decoding process. The decoding process is processed step by step in reverse by the encoding process. XOR processing is performed according to the mapping table to restore the RS code, and then the RS code is used for error correction to ensure that existing information of each sequence is accurate. Restore the BASIC code sequence. For each group of data information, decode according to the BASIC decoding algorithm.

Supplementary Note 3. Cost calculation

In this study, the cost of practical implementation of DNA storage in vivo was $0.001 per base, which was consisted of four parts: DNA synthesis, the DNA library was synthesized from Twist Bioscience and CustomArray, and the cost about $ 0.0009 per base; Assembly, this part was contained PCR and assembly, the cost around $ 58 during an experiment; Transformation, the cost of 509 oligos and 11520 oligos was $ 9 and $ 90, respectively; Recovery, which was mainly included plasmid extraction, enzyme digestion and sequencing.

Supplementary Note 4. The bioinformatic statistical analysis.

We stitched the reads pair by using PEAR to get the sequenced reads.

The sequenced reads were aligned with the actual sequences (synthesized by Twist Bioscience and CustomArray) by basic sequence alignment program (BLAST). The coverage and number could be achieved by Valid_Coverage_Number.pl. The frequency was calculated via the number dividing by total number of actual sequences. Then the distribution of number of reads per each actual sequence was displayed (Fig. 4b, Supplementary Figs 19, 22 and 23).

Valid DNA sequence named payload obtained by Obtain_Payload.pl and kmer of these payload sequences were analyzed by kmer.pl (Supplementary Figs 20 and 21).

The number of each sequence of the sequenced reads could be achieved by Valid_Coverage_Number.pl. The oligo frequency was obtained through the number of each sequence dividing by the sum of these numbers and the distribution of oligo frequency could be displayed (Fig. 3b and 3d, Supplementary Figs. 14 and 17). The Gini coefficient was calculated by R (Fig. 3e, Supplementary Figs. 15 and Supplementary Table 2).

Supplementary Note 5. Genome blast

Genomic contamination rate (%). The sequences with high similarity to the actual sequences were removed by unmatch_test.pl. The remaining sequences were aligned with the genomic sequences of DH10β (from the competent cell used in this work) by BLAST to obtain the unmatched sequence reads (namely genomic contamination reads), and genomic contamination rate was calculated via the unmatched sequence reads dividing by the total sequenced reads (Genomic contamination reads% of Table 1). In this work, the default threshold set at blast is e-value 10^{-6}. The smaller the e-value, the higher the similarity according to NCBI. At the same time, the actual sequences were also aligned with the genomic sequences by BLAST (e-value 10^{-6}), but there was no output. Then the actual sequences were aligned with the genomic sequences by blast on NCBI, the output result was: No significant similarity found.

Supplementary Note 6. Calculating error rate and dropout (%).

All sequenced reads were aligned with the actual reference sequences by BLAST to screen out the reads with errors containing substitution, insertion, and deletion (hereinafter referred to as "errors") on the payload of a single sequence. The number of reads with an error, two errors, three errors, ……, ten errors, more than ten errors in individual sequences were counted in detail by Mismatch_Analysis.pl and Gap_Analysis.pl, and the frequency were calculated through the number of these reads dividing by the total number of noisy reads (Fig. 2c, Table 1, Supplementary Fig. 11).

Dropout was calculated by Vaild_Coverage_Number.pl, Random_Access.pl and Dropout.pl (Fig. 2d, Fig. 3c, Supplementary Figs. 12, 13, 18, 24). According to our encoding strategy which allows a maximum of 4 DNA sequences to be lost or corrupted in each group (each group contains 256 DNA sequences), the redundancy can be calculated ideally as: 4/256=1.56%.

Supplementary Note 7. Calculating of storage size

The data of Fig. 4c was calculated from corresponding references, the detail was shown as follow: In 2007, Nozomu Yachie et al. has been inserted redundantly oligonucleotide (C1, C2, C3 and C4) into multiple loci of the *Bacillus subtilis* genome. The size of C1was 64 nt, C2, C3 and C4 were 62 nt, respectively. We calculated the total base is 0.25 Kbps.; In 2017, Seth L Shipman et al. has been encoded images and a short movie into the genomes of a population of living bacteria. The short movie was encoded by five frames of Eadweard Muybridge's Horse in Motion. Each Frame was represented by a unique oligo set of 104 protospacers, and each protospacer included 28 bases. We calculated the total base is 14.56 Kbps. In 2019, Jian Sun et al. has been stored a poem of "Snow" in *E. coli*, *yeast* and *Arabidopsis*. The sequence of was "Snow" was 2448 base (2.448 Kbp).
