# Supplementary material for: A mixed culture of bacterial cells enables an economic DNA storage on a large scale
Source: Commun Biol. 2020 Jul 31;3:416. doi: 10.1038/s42003-020-01141-7 (PMC7395121; doi:10.1038/s42003-020-01141-7)
Supplement: Supplementary file 2 — Description of Additional Supplementary Files [file 42003_2020_1141_MOESM2_ESM.pdf]

Description of Additional Supplementary Files

File Name: Supplementary Data File
Description: This excel document contains all primary source data used for preparing Figs. 2b, 2c, 2d, 3b, 3c, 3d, 3e, 4, Table 1 and Supplementary Figs. 11, 12, 13, 14, 15 ,17, 18, 19, 20, 21, 22, 23, 24 and Supplementary Table 2, 3.
